# The Influence of Solid Organ Transplant on Inpatient Complications, Length of Stay, and Hospital Costs in Reverse Shoulder Arthroplasty Patients

**DOI:** 10.7759/cureus.56334

**Published:** 2024-03-17

**Authors:** Garrett Sohn, Dang-Huy Do, Senthil Sambandam, Alison Cabrera, Michael Khazzam

**Affiliations:** 1 Orthopedic Surgery, University of Texas Southwestern Medical Center, Dallas, USA

**Keywords:** costs, matched, length of stay, complications, organ transplant, reverse shoulder arthroplasty

## Abstract

Introduction: With innovations in transplant medicine and longer life expectancies in solid organ transplant (SOT) recipients, the incidence of shoulder arthroplasty is predictably rising in this population. Reverse shoulder arthroplasty (RSA) has become increasingly popular due to advances in prosthetic design with expanded indications. While previous studies have examined shoulder arthroplasty in SOT patients, information specifically related to RSA patients is largely unexplored. We aim to analyze the demographics and characteristics of SOT patients who have undergone RSA while assessing inpatient complication rates, length of stay (LOS), and hospital costs in these patients compared to a matched cohort of non-transplant patients.

Methods: The National Inpatient Sample (NIS) Database was utilized to identify all patients undergoing RSA from 2016 to 2019. We generated propensity-matched groups based on pre-operative variables (diabetes, tobacco use, sex, age, and obesity) to compare complications, LOS, and inpatient costs between the SOT and control groups. T-tests and Chi-squared tests were performed where appropriate and odds ratios were calculated.

Results: We identified 59925 patients who underwent RSA. Among those, 59769 patients (99.7%) did not have a SOT and 156 patients (0.26%) had a history of SOT. Patients in the SOT group were younger than the control group (67.0 versus 71.4 years, p<0.001). The SOT group were more likely males compared to the control group (53.8% versus 39.3%, p<0.001). Following 1:1 matching, there were 156 patients in each group. The SOT group had a higher risk of acute renal failure (ARF) compared to the control group (OR 9.41, 95% CI (2.13-41.49), p<0.001). The LOS (p<0.001) and inpatient costs (p<0.001) were higher in the SOT group.

Conclusion: For RSA, SOT patients are younger and more likely male compared to those without SOT. Inpatient medical and surgical complications are similar between SOT and non-SOT patients, except SOT patients have a higher risk of ARF. SOT patients tend to have longer LOS and higher inpatient costs than non-SOT patients.

## Introduction

Solid organ transplant (SOT) is the definitive treatment for end-stage disease of the heart, lung, kidney, and liver [1]. Recent advances in immunotherapy and management of medical comorbidities have afforded increased longevity of transplanted organs as well as their recipient patients [2,3]. In 2021, the number of all donor types reached an all-time high of 41356 [4]. With longer life expectancies and associated musculoskeletal pathologies in transplant recipients, the demand for arthroplasty is predictably rising in this population as well [5]. Similarly, due to advances in surgical technique and prosthetic designs, the incidence of reverse shoulder arthroplasty (RSA) has grown exponentially [6]. RSA’s expanded indications for arthritis and fractures mean more elderly patients are likely to undergo RSA [7]. Many of these patients may have a history of SOT and understanding the implications of SOT on the medical and surgical safety of RSA in these patients is important. Because of the expanded indications of RSA compared to traditional anatomic total shoulder arthroplasty and shoulder hemiarthroplasty, post-surgical outcomes may be different. Some studies have examined shoulder arthroplasty in SOT patients but they grouped all forms of shoulder arthroplasty into a single cohort. Information specifically related to RSA patients is limited.

A recent study by Malcolm et al. assessed a large SOT patient cohort, extracted from the Nationwide Inpatient Sample (NIS) database, who received shoulder arthroplasty [5]. The study reported a higher rate of complications, a higher rate of genitourinary complications, longer hospital length of stay (LOS), and higher inpatient costs associated with the SOT group [5]. A separate study by Rizk et al. assessed functional outcomes, complications, hospital LOS, blood transfusion rates, and readmission rates between SOT and control groups receiving shoulder arthroplasty [8]. The study reported 90-day higher readmission rates, medically related complications, and transfusion requirements in the SOT group; however no difference in LOS, infection, or surgical complications [8]. Few additional studies have published data on shoulder arthroplasty in organ transplant patients as well, including a systemic review by Patel et al, which comprised only 71 all-type shoulder arthroplasties [9-13]. However, examination of this topic specifically among RSA patients remains largely unexplored. 

Objectives

The purposes of this study are (1) to analyze demographics and characteristics of SOT patients undergoing RSA; (2) to determine the medical and surgical perioperative complications of RSA in SOT patients compared to a matched cohort of non-transplant patients; (3) to assess resource-use outcomes including inpatient LOS and hospital costs in SOT and non-SOT patients undergoing RSA.

## Materials and methods

We collected patient information using the NIS database, which is part of the Healthcare Cost and Utilization Project (HCUP). It includes inpatient hospital stays, including clinical and resource use information, derived from hospital billing data and discharge records submitted to statewide organizations in the United States. The statewide data organizations are from 48 states covering 97% of the United States population. It is the largest inpatient care database in the country and draws from a stratified 20% sample of discharges from community, public, and academic hospitals. Information from the NIS database is de-identified and available to the public; therefore this study was exempt from Institutional Review Board approval. Inclusion criteria were all patients between January 2016 and December 2019 who had an RSA and a history of SOTs based on the International Classification of Diseases, Tenth Revision (ICD-10) codes. 

All procedures, comorbidities, and complications were identified using ICD-10 codes (Table 1). The ICD-10 procedural codes for patients who underwent RSA were “0RRK00Z” and “0RRJ00Z.” The SOT patients underwent were either heart, lung, liver, or kidney transplants. Demographic data included age and sex, which were provided directly by the NIS database, while comorbidities collected included diabetes, obesity, and tobacco use. We chose these variables as they are likely to increase complications in arthroplasty patients, and we wanted to mask their effect on the impact of organ transplants on complications, LOS, and hospital costs. We obtained data on the most common complications of RSA from the NIS database. The medical complications collected were deep vein thrombosis (DVT), pulmonary embolism (PE), pneumonia, myocardial infarction (MI), acute renal failure (ARF), and mortality. The surgical complications collected were periprosthetic fracture, infection, wound dehiscence, dislocation, mechanical complication, and blood transfusion requirement. Inpatient stay outcome measures were LOS and total inpatient costs, which were pre-computed and provided directly by the NIS database.

**Table 1:** ICD-10 codes used RSA, reverse shoulder arthroplasty; MI, myocardial infarction; ARF, acute renal failure; DVT, deep venous thrombosis

RSA procedural codes	Organ transplant codes	Comorbidities codes	Medical complications codes	Surgical complications codes
0RRK00Z, 0RRJ00Z	Z940, Z941, Z942, Z943, Z944	Diabetes without complications (E119); tobacco related disorder (Z87891); Obesity (E660, E6601, E6609, E661, E662, E668, E669, Z6830, Z6831, Z6832, Z6833, Z6834, Z6835, Z6836, Z6837, Z6838, Z6839)	ARF (N170, N171, N172, N178, N179); MI (I2101, I2102, I2111, I2113, I12114, I12119, I2121, I12129, I21A1); blood loss anemia (D62); pneumonia (J189, J159, J22); blood transfusion (30233N1); pulmonary embolism (I2602, I2609, I2692, I2699); DVT (I82401,I82402, I82403, I82409, I82411, I82412, I82413, I82419, I82421, I82422, I82423, I82429, I82431, I82432, I82433, I82439, I82441, I82442, I82443, I82449, I82491, I82492, I82493, I82499, I824Y1, I824Y2, I824Y3, I824Y9, I824Z1, I824Z2, I824Z3, I824Z4)	Periprosthetic fracture (M9712XA M9711XA, T84010A, T84011A, T84012A, T84013A, T84018A, T84019A, M9665, M96661, M96662, M96669, M96671, M96672, M96679, M9669, M9701XA, M9702XA); periprosthetic dislocation (T84020A, T84021A, T84022A, T84023A, T84028A, T84029A); periprosthetic mechanical complications (T84090A, T84091A, T84092A, T84093A, T84098A, T84099A); periprosthetic Infection (T8450XA, T8451XA, T8452XA, T8453XA, T8454XA, T8459XA); superficial SSI (T8141XA); deep SSI (T8142XA); wound dehiscence (T8130XA, T8131XA, T8132XA)

The IBM SPSS Statistics for Windows, Version 27.0 (IBM Corp; Armonk, NY, USA) was used to perform statistical analysis. Descriptive analyses were done for patient demographic information. Both an unmatched analysis and a matched analysis were performed. Using the pre-operative variables (diabetes, age, sex, tobacco use, and obesity), we performed a 1:1 propensity-matched analysis, yielding a SOT group and a non-SOT group, to compare inpatient medical and surgical complications, LOS, and hospital costs. Calipers and ratios were not utilized but post-match sensitivity analyses were done to ensure there were no differences between the two groups’ baseline demographic and pre-operative variables. T-tests and Chi-squared tests were used for numerical and binomial variables, respectively. For incidences less than five, Fischer exact tests were performed. For inpatient medical and surgical complications, LOS, and hospital costs, odds ratios with 95% CI were calculated as a ratio of the incidence between the SOT group and the non-SOT group. Statistical significance was set at p<0.05.

## Results

Demographics of SOT patients

Using the NIS database, we identified 59925 patients who underwent RSA. Among those, there were 59769 patients (99.7%) without a history of SOT and 156 patients (0.3%) with a history of SOT. There were 85 (55%) patients who had a kidney transplant, 38 (24%) had a liver transplant, 19 (12%) had a heart transplant, and 14 (9%) had a lung transplant.

The SOT group was significantly younger than the non-SOT group (67.0 years versus 71.4 years of age, p<0.001). The SOT group were more likely males than the non-SOT group (53.8% versus 39.3% males, p<0.001). There was no difference in tobacco use disorder, diabetes, or obesity percentage between the two groups (Table 2).

**Table 2:** Patient demographics of unmatched cohort P≤0.05 is statistically significant. SOT, solid organ transplant

	SOT group (n=156)	Non-SOT group (n=59769)	P-value
Mean age (standard deviation) in years	67.0 (7.1)	71.4 (8.6)	<0.001
Male	84 (53.8%)	23542 (39.3%)	<0.001
Diabetes without complications	24 (15.3%)	8633 (14.4%)	0.73
Tobacco use disorder	20 (12.8%)	9624 (16.1 %)	0.26
Obesity	23 (14.7%)	11941 (19.9%)	0.10

Unmatched analysis of inpatient complications, LOS, and costs

An unmatched analysis of medical complications revealed that the SOT group is at significantly higher risk of DVT, PE, and ARF (Table 3). The odds of DVT were almost 26-fold higher in the SOT group and the risk of PE was roughly 10 times higher in the SOT group. Analysis of surgical complications revealed no significant difference between both groups with respect to infection, wound complications, fractures, or mechanical complications (Table 3).

**Table 3:** Outcomes in unmatched cohorts P≤0.05 is statistically significant. ***The HCUP data use agreement precludes publishing values between one to 10. LOS, length of stay; PE, pulmonary embolism; MI, myocardial infarction; ARF, acute renal failure; HCUP, Health Care Utility Project; DVT, deep venous thrombosis; SOT, solid organ transplant

	Events in SOT group	Events in non-SOT group	Odds ratio	95% CI	P-value
Number of patients	156	59769	NA	NA	NA
LOS (days)	2.61 (SD-2.73)	1.90 (SD-2.03)	NA	NA	<0.001
Total hospital charges (dollars)	$87621.44 (SD-$60236.22)	$79667.33 (SD-$48748.35)	NA	NA	0.02
ARF	17 (10.9%)	1317 (2.2%)	5.42	3.27-9.00	<0.001
MI	0	29 (0.04%)	NA	NA	NA
Blood loss anemia	25 (16.0%)	6111 (10.2%)	1.67	1.09-2.57	0.02
Pneumonia	*** (1.3%)	225 (0.4%)	3.43	0.84-13.95	0.06
Blood transfusion	*** (4.5%)	1170 (1.9%)	2.35	1.10-5.03	0.02
PE	*** (1.3%)	73 (0.1%)	10.62	2.50-43.65	<0.001
DVT	*** (1.9%)	45 (0.07%)	26.02	8.00-84.60	<0.001
Peri-prosthetic fracture	*** (0.6%)	132 (0.2%)	2.91	0.40-20.97	0.26
Peri-prosthetic dislocation	*** (2.6%)	996 (1.6%)	1.55	0.57-4.19	0.38
Peri-prosthetic mechanical complications	*** (1.3%)	750 (1.2%)	1.02	0.25-4.13	0.97
Peri-prosthetic infection	*** (0.6%)	344 (0.5%)	1.11	0.15-7.98	0.91
Wound dehiscence	0	19 (0.03%)	NA	NA	NA
In-hospital mortality	0	39 (0.06%)	NA	NA	NA

The mean total inpatient costs were higher in the SOT group ($87,621.44±$60,236.22) compared to the non-SOT group ($79,667.33±$48,748.35; p=0.02). The mean LOS was higher in the SOT group (2.61±2.73 days) than in the non-SOT group (1.90±2.03 days; p<0.001).

Matched analysis of inpatient complications, LOS, and costs

Following 1:1 propensity matching, there were 156 patients in the SOT group and 156 patients in the non-SOT group. The SOT group had a higher risk of ARF than the non-SOT group (OR 9.41, 95% CI (2.13-41.49), p<0.001). The risks of blood loss anemia and blood transfusion were higher in the SOT group but failed to reach statistical significance. All other medical and surgical complications failed to show any statistical significance in the matched data (Table 4).

**Table 4:** Outcomes in matched cohorts P≤0.05 is statistically significant. ***The HCUP data use agreement precludes publishing values between one to 10. LOS, length of stay; PE, pulmonary embolism; MI, myocardial infarction; ARF, acute renal failure; HCUP, Health Care Utility Project; DVT, deep venous thrombosis; SOT, solid organ transplant

	Events in SOT group (156)	Events in non-SOT group (156)	Odds ratio	95% CI	P-value
Number of patients	156	156	NA	NA	NA
LOS (days)	2.61 (SD-2.73)	2.02 (SD-1.67)	NA	NA	<0.001
Total hospital charges (dollars)	$87621.44 (SD-$60236.22)	$53566.13 (SD-$25799.76)	NA	NA	<0.001
ARF	17 (10.9%)	*** (1.3%)	9.41	2.13-41.49	<0.001
MI	0	0	NA	NA	NA
Blood loss anemia	25 (16%)	14 (9%)	1.93	0.96-3.88	0.06
Pneumonia	*** (1.3%)	0	NA	NA	0.16
Blood transfusion	*** (4.5%)	*** (3.2%)	1.41	0.44-4.57	0.55
PE	*** (1.3%)	*** (0.6%)	2.01	0.18-22.43	0.56
DVT	*** (1.9%)	0	NA	NA	0.08
Peri-prosthetic fracture	*** (0.6%)	0	NA	NA	0.31
Peri-prosthetic dislocation	*** (2.6%)	*** (3.8%)	0.65	0.18-2.37	0.52
Peri-prosthetic mechanical complications	*** (1.3%)	*** (3.2%)	0.392	0.07-2.05	0.25
Peri-prosthetic infection	*** (0.6%)	*** (0.6%)	1	0.06-16.1	1.00
Wound dehiscence	0	0	NA	NA	NA
In-hospital mortality	0	*** (0.6%)	NA	NA	0.32

The mean total inpatient costs were higher in the SOT group ($87,621.44 ± $60,236.22) than in the non-SOT group ($53,566.13 ± $25,799.76; p < 0.001). The mean LOS was higher in the SOT group (2.61 days ± 2.73 days) than in the non-SOT group (2.02 days ± 1.67 days; p < 0.001).

## Discussion

Advances in both surgical techniques and immunotherapies have yielded a large population of transplant recipients who are susceptible to the same orthopedic conditions as the general population. Although certain conditions, such as osteonecrosis and infection, may be more common in the SOT patient, fractures and osteoarthritis remain prevalent in any patient [14,15]. Innovations in RSA prosthetic design and surgical techniques have allowed for expanded RSA indications for shoulder arthritis and fracture treatment, compared to traditional shoulder arthroplasties [7]. Because many patients who will be considered for RSA will have a history of SOT, understanding the influence of SOT on medical and surgical outcomes along with resource use outcomes is important. At present, there is little orthopedic literature that has analyzed RSA in SOT patients. To date, our study represents the largest analysis of SOT patients who have undergone RSA specifically.

Regarding demographic data of each cohort, this study found that SOT patients were younger and more likely male than non-transplant patients undergoing RSA. However, this study did not find a significant difference in baseline rates of uncomplicated diabetes mellitus, tobacco use, or obesity. This contrasts with the previous study by Malcolm et al., which reported higher rates of multiple comorbidities, complicated and uncomplicated diabetes, hypertension, deficiency anemia, congestive heart failure, valvular heart disease, and coagulopathy for transplant patients [5]. Previous literature suggests SOT patients are more fragile and medically complex than the general population [5,10]; however, our demographic data do not yield any statistical significance in regard to these baseline comorbidities.

It is important to highlight potential associated medical and surgical complications with elective RSA in transplant patients. Prior to matched cohort analysis, this study identified significantly higher rates of ARF, blood loss anemia, DVT, PE, and the need for blood transfusion in the SOT cohort. Pneumonia also was noted to be higher within the transplant group as well, although this failed to reach statistical significance. After matched cohort analysis, the risk of ARF was found to be significantly higher in the SOT cohort. Previous literature has highlighted ARF as a known post-operative risk in transplant patients undergoing total hip arthroplasty [2,16]. The prior work by Malcolm et al. highlighted ARF as a significantly higher risk in their transplant patient prior to matched cohort analysis, but the risk failed to reach statistical significance after matched analysis [17]. RSA appears to be a safe procedure from the medical perspective in the short term when performed in SOT patients. These patients should be counseled that, given the available literature, they are at increased risk of ARF compared to non-transplant patients.

Interestingly, our study did not identify a significant difference in rates of periprosthetic fracture, periprosthetic infection, mechanical complications, or wound dehiscence between the transplant and control groups. Prior research has demonstrated both the increased risk of post-operative infection and periprosthetic fracture in the transplant patient after total joint arthroplasty, particularly due to the patient’s baseline immunosuppressed status and poor bone quality [15-19]. Of note, Hatta et al. analyzed a cohort of post-transplant patients on immunosuppression after shoulder arthroplasty at a mean follow-up of 39 months and reported no post-operative infections [10]. Although this complements the lack of significance found in our data, the results are mixed.

Inpatient LOS and admission costs are important surrogates of total medical costs and health care burden following arthroplasty procedures. Previous literature has reported that hospital LOS and inpatient admission costs are increased in transplant patients undergoing total joint arthroplasty compared to non-transplant patients [2,5,13,17]. Our analysis supports these findings, with SOT patients having an average LOS of 2.61 days and an average hospital cost of $87.621.40, compared to the control group LOS of 2.02 days and a cost of $53566.13. As the annual number of arthroplasties increases, notably RSA’s, particular attention should be paid to managing costs and healthcare utilization with regard to transplant patients.

Despite the large cohort of patients analyzed, this study is not without limitations. Similar to other database studies, the NIS database is limited by potential coding inaccuracies and lack of individual specifics in the clinical data since the information is de-identified. Second, this study only includes short-term inpatient complications and outcomes as it is based on discharge records reported by hospitals. This data does not capture mid-term and long-term follow-up and does not include complications or costs encountered after discharge, which may lead to underreporting these outcomes. Third, we are unable to identify the specific diagnoses leading to RSA, whether patients were on immunosuppressive medications and the costs of those immunosuppressive medications. Differences in specific diagnoses such as avascular necrosis and the type of immunosuppressive medications may potentially influence providers on the decision to proceed with surgery. Fourth, because the database captures data from community, academic, and public hospitals, there may be differences in outcomes, especially total costs and LOS, between different hospital types that could not be captured in this study. Fifth, despite propensity matching with common arthroplasty risk factors and confirming group similarity in sensitivity analyses, potential confounding factors that could have influenced our findings include, as previously mentioned, the reason for RSA, immunosuppressive medication type, and hospital type. Finally, the NIS database provides overall inpatient costs from discharge data directly, so we are unable to discern the differences in the cost of medications, diagnostic tests, and procedures between the groups. 

Strengths of this study include its large sample size, which enhances the statistical power and generalizability of our results. It is the largest known analysis of SOT patients evaluating clinical and resource use outcomes. Additionally, we controlled for potential confounding variables by performing propensity matching to create comparable groups, which strengthens the validity of our findings. We performed a thorough analysis to assess perioperative complications, LOS, and inpatient costs, which provides a comprehensive understanding of the clinical and resource use outcomes in patients undergoing RSA with SOT. Finally, while previous studies have evaluated all forms of shoulder arthroplasty together in patients with SOT, RSA has expanded indications for arthritis and fractures, so outcomes may be different than previous arthroplasties. So, this study provides novel and valuable insight into the outcomes of SOT patients with RSA specifically.

## Conclusions

For RSA, SOT patients are younger and more likely male compared to those without SOT. Inpatient medical and surgical complications are similar between SOT and non-SOT patients, except SOT patients have a higher risk of ARF. SOT patients tend to have longer LOS and higher inpatient costs than non-SOT patients. Further research is needed to elucidate long-term and functional outcomes following RSA. Regardless, with modern advances in immunotherapy, transplantation medicine, and arthroplasty techniques, the importance of further study cannot be understated.
